# Aerogel Composites Produced from Silica and Recycled Rubber Sols for Thermal Insulation

**DOI:** 10.3390/ma15227897

**Published:** 2022-11-08

**Authors:** Alyne Lamy-Mendes, Ana Dora Rodrigues Pontinha, Paulo Santos, Luísa Durães

**Affiliations:** 1 University of Coimbra, CIEPQPF, Department of Chemical Engineering, Rua Sílvio Lima, 3030-790 Coimbra, Portugal; 2 University of Coimbra, ISISE, Department of Civil Engineering, Rua Luís Reis Santos, 3030-788 Coimbra, Portugal

**Keywords:** rubber-silica aerogel, recycled rubber sol, fibers reinforcement, thermal superinsulation

## Abstract

Hydrophobic rubber-silica aerogel panels (21.5 × 21.5 × 1.6 cm^3^) were fabricated from silica and rubber sols and reinforced with several fiber types (recycled tire textile fibers, polyester blanket, silica felt, glass wool). A recycled rubber sol was prepared using peracetic acid and incorporated for the first time in TEOS-based sol-gel chemistry. The composites exhibited good thermal stability up to 400 °C and very low thermal conductivity, in the superinsulation range when using polyester fibers (16.4 ± 1.0 mW·m^−1^·K^−1^), and of 20–30 mW·m^−1^·K^−1^ for the remaining fibers. They could also endure cyclic compression loads with near full recovery, thus showing very promising properties for insulation of buildings.

## 1. Introduction

One of the most critical issues in modern society is the constant increase of waste [1]. Currently, over 1.6 billion new tires and around 1 billion of waste tires are generated worldwide every year [2]. In the last decade, there has been a growth in the number of tires being discarded as end-of-life tires (ELTs) [1], which leads to serious environmental problems. In order to improve the waste management practices, the European legislation has established a priority order for dealing with wastes, from the most preferred option of reduction, followed by reuse, recycling, and energy production, to the least preferred option of disposal [3,4,5]. However, significant difficulties are associated with the recovery and recycling of used tires, due to their composition, complex structure, and chemical stability [6]. The main component of the tires (70–80% of the total mass) is vulcanized rubber, and its disposal is an issue, as it is non-biodegradable and cannot be reprocessed in a simple way like the thermoplastics, remaining on the landfills for uncountable years [1,6,7]. This is also valid for the tire-reinforcing fibers, as they are made by highly thermally stable materials and, when recovered from tires, they result in very short fibers [1].

To engage in more environmentally friendly solutions, and comply with more restrict legislations, new methods for the treatment or reuse/recycling of the basic components of ELTs are being developed, some of them provided products of high added value. Dwivedi and co-authors [7] were able to use the carbon-rich solid (RCB) fraction, from the pyrolysis of waste tires as a partial substitute to conventional carbon black (CB) in natural rubber-based conveyor belts cover composites. The mechanical properties of the RCB-reinforced composites were lower than the material obtained with conventional CB. However, when a combination of RCB and commercial CB was used, these limitations could be overcome, and the impact in the mechanical properties is acceptable. This partial replacement reduces the cost of rubber compounds and provides a sustainable recycling option of waste tires.

Another approach was developed by Passaponti et al. [8], in which the authors were able to recover and valorize the chars, the least valuable byproduct of waste tire pyrolysis. They were able to convert this material into highly efficient catalysts for the Oxygen Reduction Reaction (ORR), with a simple heat treatment. The sample prepared at 450 °C displayed the maximum catalytic activity, with a conversion efficiency of O_2_ to H_2_O above 85%. The developed material has potential to be applied in alkaline fuel cells and metal-air batteries. 

Wang et al. [9] were able to directly convert waste tires into 3D graphene, by raising the pyrolytic temperature to 1000 °C and using an active K vapor to induce carbon atom rearrangement. The final material displays a well-defined porous gradient and high electrical conductivity (18.2 S·cm^−1^). When applied as supercapacitor electrode, graphene exhibits an excellent capacitive behavior, with almost no degradation after 10,000 cycles. This work provides a new consideration for the development of more high-value products from waste tires.

Another possibility to obtain high value products is the synthesis of aerogels from waste tires, as presented by Thai et al. [10]. The authors were able to obtain aerogels from recycled car tire textile fibers through the freeze-drying method, using polyvinyl alcohol and glutaraldehyde as crosslinkers of the rubber granules attached to these fibers. This aerogel showed densities as low as 35 kg·m^−3^, porosity up to 96%, thermal and sound insulation properties with thermal conductivities between 35 and 47 mW·m^−1^·K^−1^, and a noise reduction coefficient of 0.41. In addition to that, the aerogel also had an oil absorption capacity of 19.3 g·g^−1^, showing the versatility of the developed material. 

Motivated by the environmental problems caused by ELTs and the current potential markets for products obtained with recycled/reused materials, our goal was to develop fiber-reinforced rubber-silica aerogel composites. As it is known, rubber is a flexible material, hydrophobic and extremely stable. Moreover, recycled tire rubber is a cheap raw material. Thus, the introduction of recycled rubber into the aerogels not only contributes to improving their mechanical properties and reducing shrinkage during drying, but also allows the reduction of the aerogels cost and the environmental carbon footprint. 

The present work elucidates the synthesis of aerogel composites involving a two-step acid–base catalyzed hydrolysis and condensation of tetraethylorthosilicate (TEOS) in the presence of recycled tire rubber in a sol phase. For the first time, a rubber sol, using rubber from ELTs, was produced, and incorporated into silica sols, i.e., the rubber phase was completely mixed as a sol phase and not as granules. This manuscript also presents the effect of different fibers, organic and inorganic, in the composites’ final properties for the same synthesis protocol. The presented manufacturing procedures were further developed to obtain large panel samples, with a size of 21.5 × 21.5 × 1.6 cm^3^, keeping the shrinkage of the gels negligible during ambient pressure drying. The application of this type of drying, instead of the costlier and more complex supercritical drying, was possible due a due to the combination of rubber-fibers-HMDSO/TMCS silylation and reinforcement strategies. The used approach for the manufacturing of the resultant composites has never been reported in the open literature. The final aerogel composites (small and large samples) were characterized regarding their chemical, physical, structural, and thermomechanical properties. Due to their thermal superinsulation ability (keeping the best feature of pure silica aerogels) and improved mechanical resistance, the aerogel composites not only promote the recycling of waste tires, but also present a huge potential for reducing the price of aerogels and unlock the widening of thermal insulation applications. 

## 2. Materials and Methods

### 2.1. Materials

Tire rubber (diameter < 0.8 mm), ethanol (absolute, C_2_H_5_OH, Fluka), peracetic acid (38–40%, CH_3_CO_3_H, Merck), tetraethylorthosilicate (TEOS; purity ≥ 99%, Si(OC_2_H_5_)_4,_ Aldrich), ammonium hydroxide (25% NH_3_ in H_2_O, NH_4_OH, Fluka Analytical), n-hexane (C_6_H_14_, purity > 95%, Fisher Chemical), hexamethyldisiloxane (HMDSO, (CH_3_)_3_SiOSi(CH_3_)_3_, purity > 98%, Acros Organics), trimethylchlorosilane (TMCS, (CH_3_)_3_SiCl, purity ≥ 98%, Sigma Aldrich), and four different fibers were used in the composite production. The fibers acted as reinforcement of composites. The first type tested, tire textile fibers, include the following three main components: (i) textile fibers (Nylon 6 and 66, Polyester, Kevlar, Rayon and Glass); (ii) natural rubber and synthetic rubber polymers attached to the fibers; and (iii) residues of steel wire. A polyester fiber blanket, a felt of silica fibers with very low bulk density (<20 kg·m^−3^) [11], and glass wool were also applied as alternative to the tire textile fibers.

### 2.2. Composite Synthesis

The first step for the composite synthesis consists of dissolving the 0.5 g of tire rubber in a 10 mL solution containing ethanol and peracetic acid, with acid concentration of 5.0 vol·%. The solution was stirred for 24 h. After this period, a black colloidal solution was obtained. 

**Figure 1:**
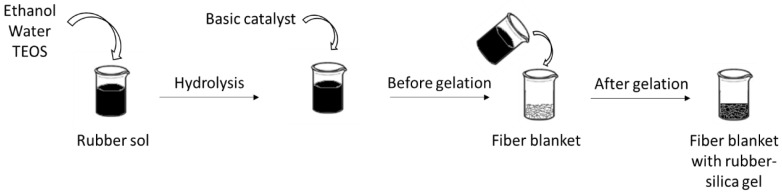
Illustration of the synthesis of the rubber-silica aerogel composite reinforced with fibers.

For the second step, the rubber colloidal solution was mixed with the silica sol. Therefore, ethanol, TEOS, and double distilled water, in a molar ratio of ethanol:Si:water = 10:1:4, were added to the rubber colloidal solution, and the mixture was stirred for 30 min. The solution was then stored in an oven at 27 °C for 24 h for the hydrolysis step. A basic solution, NH_4_OH 2.5 M, was added to the former solution and kept under strong agitation for 1 min and then poured into the mold with the fiber blanket (Figure 1). The samples were kept in the oven at 27 °C during 5 days for aging. 

Different types of fibers can be used for the composites’ reinforcement, from recycled tire textile fibers to glass and polyester fibers. In the case of recycled tire textile fibers, they were used in their pristine state and after being modified by an acid solution treatment. For this treatment, 5 g of tire textile fibers were mixed with an alcoholic solution of 2.5 vol·% of peracetic acid. The mixture was stirred for 1–2 h and then filtered. The fibers were washed two times with ethanol using the centrifuge and then dried at 60 °C in an oven.

The composite gels were unmolded and washed with ethanol and hexane at 50 °C. The samples were then subjected to a surface modification with HMDSO and TMCS in order to obtain hydrophobic aerogels able to resist shrinkage during drying. The addition of methyltrimethoxysilane (MTMS) as co-precursor could be a way to eliminate this step [12], but it is known that it generates macropores in the silica structure, which contribute to the degradation of thermal insulation ability as it reduces the Knudsen effect. The combination of the used silylating agents (TMCS and HMDSO) had two main objectives. TMCS is one of the most efficient silylating agents, although its use leads to the release of HCl. On the other hand, HMDSO needs HCl for the scission of the siloxane bond and promote reaction. The mixture of HMDSO with a small amount of TMCS not only reduces the amount of released HCl but also takes advantage of the generated acid to increase the reactivity of HMDSO, without the addition of further chemicals.

The silylating solution comprised hexane, HMDSO, and TMCS (70:20:10 volumetric percentages). First, hexane and HMDSO were mixed, and the solution was stirred for 30 min, and then the TMCS was added, and the solution was stirred for another 30 min. The samples were immersed in the silytating solution and then placed in an oven at 50 °C and kept at that temperature for 8 h. After that, the samples were kept in the silytating solution for another 48 h at a temperature between 15 and 35 °C. To dry the samples, the solution was removed, and the samples were kept in the hood for 24 h and then subjected to 100 °C for 3 h and 150 °C for 3 h.

### 2.3. Materials and Aerogels Characterization

The properties of the initial materials and final composite materials were assessed by different characterization techniques. The bulk density (*ρ*_b_) was calculated from the weight and volume of regular pieces of the samples. The chemical structure was evaluated by attenuated total reflection (ATR) Fourier-transform infrared spectroscopy (FTIR) (*FT/IR 4200, Jasco,* Tokyo, Japan), collecting the spectra between a wavenumber of 4000 and 400 cm^−1^, with 128 scans and 4 cm^−1^ of resolution. For the rubber, elemental analysis (EA) was also performed (*EA 1108 CHNS-O, Fision Instruments,* Waltham, MA, USA), in terms of C, N, H and S elements. Specific surface area (*S*_BET_) was assessed through nitrogen adsorption-desorption and the Brunauer–Emmet–Teller (BET) model (*ASAP 2000, Micrometrics,* Norcross, GA, USA). The average pore size was obtained by applying the Barrett–Joyner–Halenda (BJH) model to the desorption branch of the isotherm, complemented with pore size distribution in the range from 0.1 to 300 nm. Scanning electron microscopy (SEM) images were obtained using a *Compact/VPCompact FESEM (Zeiss Merlin,* Leipzig, Germany*)* microscope, after coating the aerogel samples with a thin gold layer by Physical Vapor Deposition, during 20 s. Thermal properties were assessed by thermal gravimetric analysis and thermal conductivity. The thermal stability of different materials was obtained by using a DSC/TGA equipment (*TGA-Q500, TA Instruments,* New Castle, DE, USA), from 20 °C to 800 °C, at a 10 °C·min^−1^ heating rate under nitrogen flow. Thermal conductivity, *k*, was measured with a *Thermal Constants Analyzer TPS 2500 S (Hot Disk,* Göteborg, Sweden*)*, using the transient plane source method with two samples maintained at 20 °C. For panel samples with dimensions of 21.5 × 21.5 × 1.6 cm^3^, the thermal conductivity was also determined using a Heat Flow Meter *HFM 436/3/1 Lambda* (EN 1946-1:1999), from *NETZSCH* (Selb, Germany), at 23 °C. The results obtained in this equipment are in close agreement with those obtained by the Guarded Hot Plate method [13]. The dynamic stiffness, *s*^’^_t_, was also measured for the most insulating panel aerogel (reinforced with polyester) following the test procedures defined in standard ISO 9052-1 [14]; the test-samples had a thickness of 1.5 cm and an area of 20.0 × 20.0 cm^2^. Static mechanical tests were also performed using an *Inspekt mini-series* equipment, from *Hegewald and Peschke* (Nossen, Germany). Uniaxial compression-decompression of the samples with a load cell of 50 N was carried out up to 25% strain, using a deformation rate of 1 mm min^−1^, providing the Young’s modulus and recovery percentage of the samples. In addition, 10 cycles of compression-decompression with a maximum strain of 10% were used to assess the recovery of the samples to cyclic loading. Finally, a destructive compressive test with a cell of 3 kN was implemented, letting the samples to be compressed up to the limit of the loading cell.

## 3. Results and Discussion

### 3.1. Rubber Dissolution

**Figure 2:**
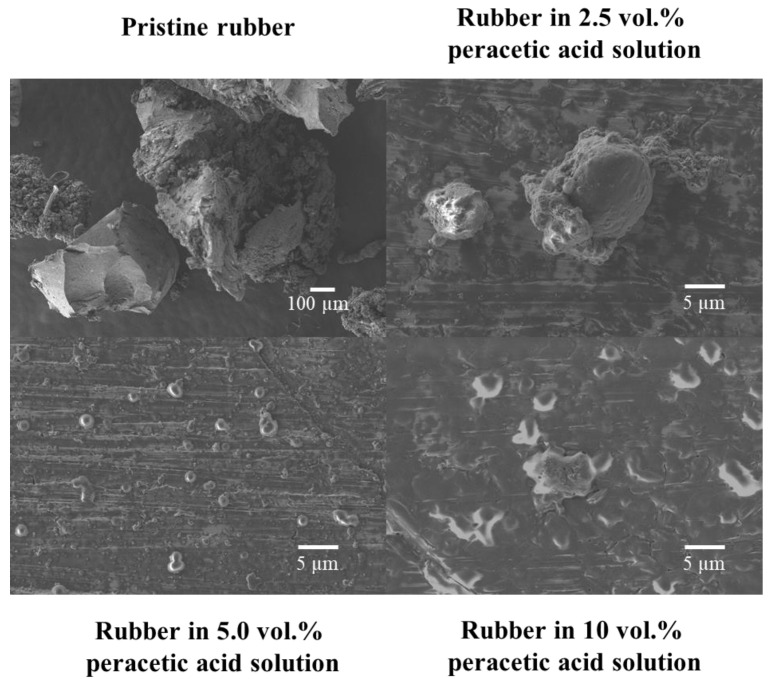
SEM images of pristine rubber and rubber submitted to different concentrations of peracetic acid solutions.

The high stability of the rubber makes it difficult to chemically bond it to the other phases of the composite. Thus, the modification of the rubber surface, or its dissolution in a solvent mixture are interesting approaches for improving the interaction between the silica matrix and the rubber. Different concentrations of peracetic acid solution can be used for obtaining a homogeneous colloidal solution. To assess the effect of the acid solution in the rubber particles, three of the tested solutions were analyzed by scanning electron microscopy (Figure 2). 

The solution with a concentration of 2.5% of peracetic solution was able to partially reduce the size of the rubber particles; however, after a few hours, it was possible to see the particles at the bottom of the solution, indicating a phase separation by their settling. When a solution of 5% of peracetic acid was used to react with the pristine rubber and obtain a colloidal solution, the best particle size distribution was obtained, with all the particles having diameter lower than 1 µm, and this solution remained stable for several days. For a concentration of 10% of peracetic acid, the observed entities have larger sizes than the ones obtained for the 5% solution. It was expected that for higher concentrations, lower sizes were going to be obtained, but this tendency was not observed in the SEM images. It is most likely that the small particles are agglomerating into large clusters, as some particles with small sizes are observed in the image (Figure 2).

### 3.2. Structural and Thermal Characterization of the Composites

The characterization of the granulated rubber (see Section S1) and the fibers (see Section S2) used as reinforcement mat were performed and the results are reported in the Supporting Information.

**Figure 3:**
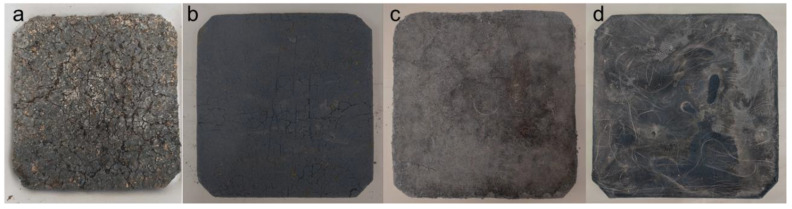
Macro-photographs of the rubber-silica aerogel composite panels reinforced with (**a**) recycled tire textile fibers; (**b**) polyester fiber blanket; (**c**) silica fiber felt; and (**d**) glass wool.

Table 1 shows key properties of the composites obtained in this work and Figure 3 the macro-photographs of the samples. 

The composites displayed very low shrinkages during the drying step (Table 1), thus keeping intact the pore structure of the gel, which contributes to the excellent insulation performance. Two main factors contribute to that. First, when fibers are added into the aerogel matrix, they can resist lateral capillary stresses developed during the drying procedure and thus act as supporting skeletons. The second factor is related to the modification of the silica matrix. After the modification step, the silica gel has a hydrophobic character (Table 1, see contact angle), which makes possible the “spring-back” effect of the matrix. During ambient pressure drying, first there is a contraction of the gel due to capillary pressure, followed by a partial recovery to its initial volume. This recovery is caused by the presence of non-condensable moieties/non-polar groups grafted in the silica matrix surface [15,16,17]. With the simultaneous use of HMDSO and TMCS in the modification step, a great part of the OH groups are converted to O-Si-(CH_3_)_3_ [15]. The CH_3_ groups on the surface repel each other when the pores tend to reduce size during drying, leading to the “spring-back” effect (reversible shrinkage) [15,16,17]. 

**Scheme 1:**
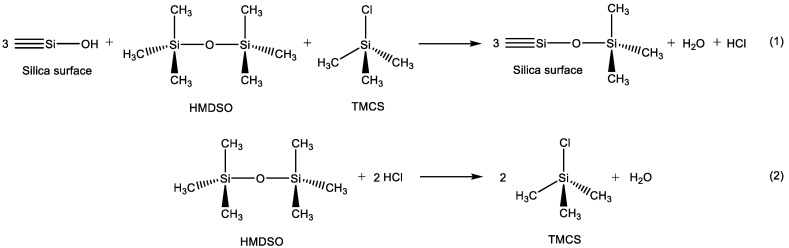
Reactions of HMDSO and TMCS with silica surface (1) and reaction of HMDSO with HCl forming TMCS (2).

The individual reactivities of HMDSO and TMCS are complementary; TMCS enhances the reactivity of HMDSO, since the HCl needed for the cision of the HMDSO is formed during the reaction of TMCS with silica pendant hydroxyl groups. The occurring reactions are displayed in Scheme 1. As these chain reactions occur, they enhance the surface modification and lead to the formation of an aerogel matrix with uniform structure and low density [15,18], as observed in the composites here developed. 

Comparing the composites in Table 1, similar values of bulk density were obtained for the composites synthesized with the polyester fiber blanket, silica fiber felt, and glass wool, with these being lower than the values obtained for the composites with recycled tire textile fibers, which may contribute to better insulation properties. The differences are probably due to the densities of the fibers themselves, with the recycled tire textile fibers having a higher bulk density, 144.2 kg·m^−3^_,_ than the other fibers. 

Even though a higher value was obtained for the composite with recycled tire textile fibers, the materials have densities in the same range of other fibers-reinforced silica aerogel composites dried in ambient pressure conditions [17,19,20,21,22]. In aerogels, the density has a strong influence in the thermal conductivity of the samples [23,24], with most of the relevant superinsulating SiO_2_ aerogels commercially available having densities between 80 and 200 kg·m^−3^ [25]. As all composite materials have densities in this range, their thermal conductivity was also assessed (Table 1). 

**Figure 4:**
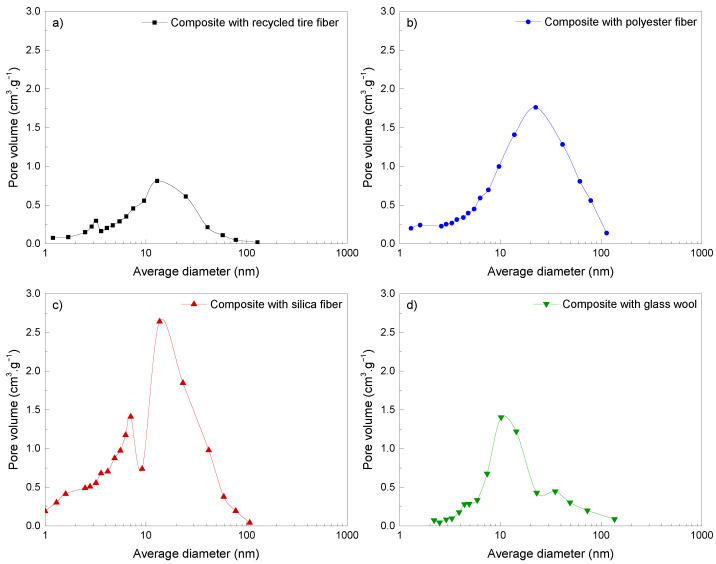
Pore size distribution for the rubber-silica aerogel composites reinforced with (**a**) recycled tire textile fibers; (**b**) polyester fiber blanket; (**c**) silica fiber felt; and (**d**) glass wool.

The samples that exhibited lower densities and thermal conductivities are also those that show higher specific surface area (Table 1), which was expected. In the case of the samples reinforced with (i) recycled tire fibers and (ii) glass wool, the lower specific surface area may be related to: (i) the existence of non-porous rubber granules and other impurities attached to the fibers and the weak interaction of the fibers with the silica matrix, both creating large interstices in the material; (ii) the non-organization of the fibers in a mat and consequent non-regular distribution of fibers, which may lead to larger voids in the matrix. The average pore sizes (in the mesopores range) show similar values as well as the pores size distributions (Figure 4), but with this technique, it is not possible to evaluate the macropores range. It should also be noted that the extent of mesoporosity is much higher in the aerogels reinforced with polyester and silica fiber blankets, which equally explains their superior performance in the discussed properties. We will return to this subject when discussing SEM analysis.

The measuring of the thermal conductivity is crucial to establish the possibility of applying the developed composites as thermal insulator materials. The aerogel composite with recycled tire textile fibers has higher values of thermal conductivity than the other ones. This was expected since a higher density leads to an increase of the contribution of the solid component in the thermal conductivity [17]. 

Even though the recycled rubber presents a high thermal conductivity (see Section S1—Supporting Information), its addition into the silica sol did not cause an increase in the thermal conductivity of the final aerogel when compared to pure silica aerogels. Other advantages were already indicated in the Introduction which show that we found a successful strategy to create materials that follow a circular economy approach [26]. The pure silica aerogel, TEOS-based matrix with the same modification procedure as the composites, has a thermal conductivity of 24.67 ± 0.14 mW·m^−1^·K^−1^, while the rubber-silica aerogel (addition of rubber into the TEOS sol and modified with HMDSO/TMCS) exhibits a value of 24.82 ± 0.05 mW·m^−1^·K^−1^, when measured by Hot Disk transient method. The similar values of both aerogels indicate a good interaction between the two phases to form the three-dimensional network, which was later confirmed by SEM images. 

All the composites disclosed in Table 1**.** display similar or even lower thermal conductivities than the typical building insulation materials used in walls such as fiberglass (33–40 mW·m^−1^·K^−1^), rockwool (37 mW·m^−1^·K^−1^), polyethylene (41 mW·m^−1^·K^−1^), expanded polystyrene (37–38 mW·m^−1^·K^−1^), extruded polystyrene (30–32 mW·m^−1^·K^−1^), and cellulose (46–54 mW·m^−1^·K^−1^) [27]. The lowest value was achieved by polyester fiber-reinforced rubber-silica aerogel composite that has a thermal conductivity lower than 25 mW·m^−1^·K^−1^, even when measured by the Hot Disk method, being classified as a superinsulating material [25]. The composites obtained with the inorganic fibers, silica felt, and glass wool, presented slightly higher values than the samples with PET fibers, and this can be once again explained by the variations observed in their densities. Different authors verified that the dependency of thermal conductivity with the bulk density typically has a U-shape [23,28,29,30,31,32], with higher densities favoring the heat transfer in the solid component, while the gaseous thermal conductivity has a higher effect for samples with lower densities, as the presence of macropores do not contribute to the Knudsen effect [23,32].

**Figure 5:**
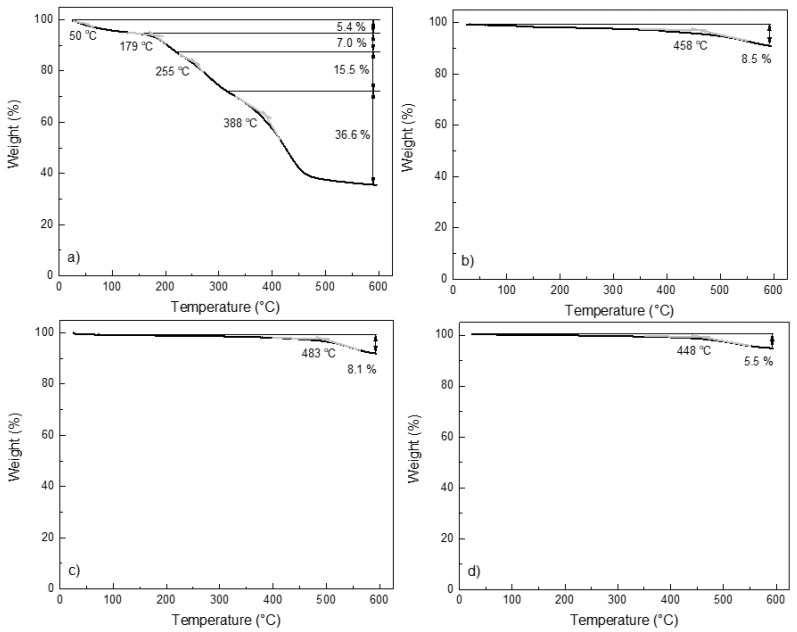
TGA of the rubber-silica aerogel composites reinforced with (**a**) recycled tire textile fibers; (**b**) polyester fiber blanket; (**c**) silica fiber felt; and (**d**) glass wool.

To estimate the thermal stability of the composite materials, the samples were submitted to a thermogravimetric analysis from 20 °C to 600 °C (Figure 5). For the composite with recycled tire textile fibers, a significant weight loss was detected, Figure 5a, with four phenomena being observed. The first weight loss starts right at room temperature (20–25 °C) and is due to adsorbed water and residual solvents/byproducts of the synthesis procedure (*T*_onset_ = 50 °C). The second phenomenon, onset temperature of 179 °C, can be attributed to the loss of structural hydroxyl groups of TEOS precursor. The third and fourth weight losses (*T*_onset_ = 255 °C and 388 °C, respectively) are mainly due to the thermal degradation of the composites’ fibers (see Section S2.1—Supporting Information and Figure S2b), first fibers such as rayon and later the polyester and polyamide fibers. However, the last phenomenon also has the contribution of the decomposition of methyl groups attached to the silica surface after the modification with HMDSO and TMCS [17,33].

For the composites made with polyester fibers, only a small weight loss was observed, around 8.5% (Figure 5b). The onset temperature was 458 °C, much higher than the values obtained for the composite with recycled tire textile fibers. Very similar results were obtained for the samples obtained with the silica fiber felt and glass wool (Figure 5c,d), with these composites presenting weight losses of 8.1% and 5.5%, and onset temperatures of 483 °C and 448 °C, respectively. The weight loss observed for these three types of composites can be attributed to the thermal decomposition of the silica surface’ methyl groups from silylation [17,33], as also verified in the composite material obtained with recycled tire textile fibers. In all cases, after the degradation of -CH_3_ surface groups, it is expected that the materials lose their hydrophobic character [17,34]. 

**Figure 6:**
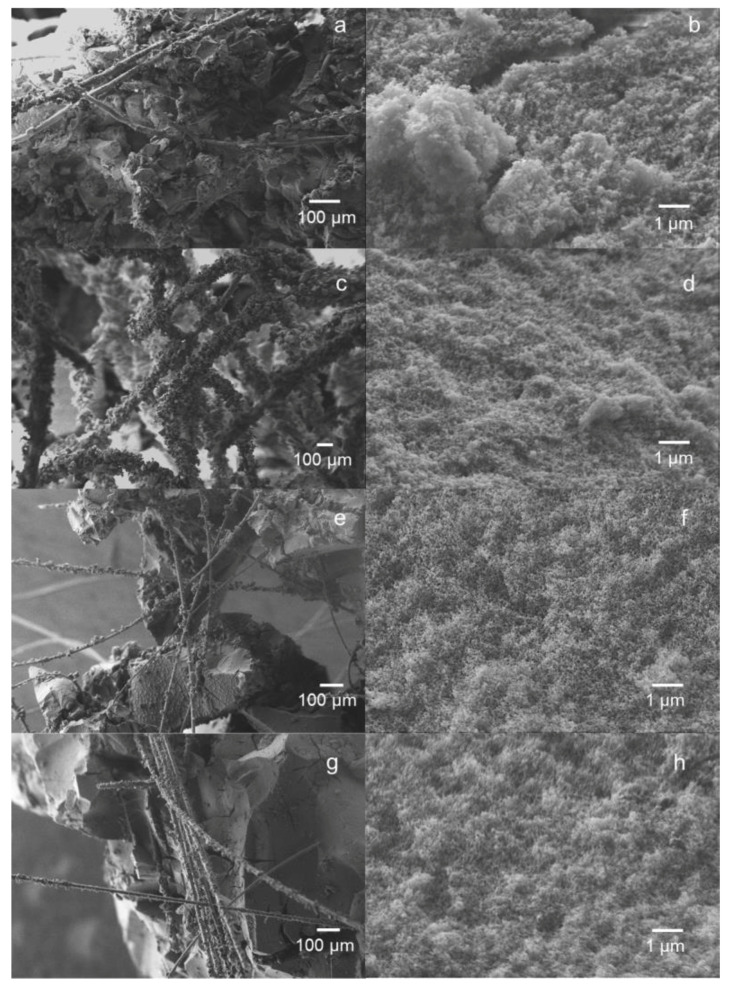
SEM images of rubber-silica composites reinforced with (**a**,**b**) recycled tire textile fiber, (**c**,**d**) polyester fiber blanket, (**e**,**f**) silica fiber felt, and (**g**,**h**) glass wool. (**a**,**c**,**e**,**g**) Interaction of the silica phase with the fibers; (**b**,**d**,**f**,**h**) rubber and silica matrix.

The significant difference in the thermal degradation between the composite with recycled rubber fibers and the other developed composites is probably due to the different interaction of the rubber-silica aerogel with the fibers, as observed in the SEM images (Figure 6). For the composite synthesized with recycled tire textile fibers, most of the fibers are still exposed (Figure 6a), indicating that the fibers do not interact significantly with the aerogel, with a clear separation between both phases. For the remaining materials, the aerogel was able to cover the fibers partially/completely (Figure 6c,e,g), increasing their thermal stability.

For the composites with the silica fiber felt and glass wool (Figure 6e,g), some degree of interaction occurs between the two phases, with some of the fibers being covered by the aerogel while other remain exposed. For these two composites the interaction was not as good as in the case of polyester fibers (Figure 6c), in which it is easily observed that the aerogel grew around the fiber following the fibers’ shape. 

Regarding the aerogel phase, all the samples show similar microstructures (Figure 6) with an interconnected three-dimensional aerogel matrix. Thus, even though the interaction between both phases is different, it can be concluded that the type of fibers used does not affect the formation of the porous structure of the matrices. It was also verified that the presence of colloidal rubber did not prevent the formation of the network or interfered in the typical silica aerogel structure, as all the samples present similar structures observed in other modified TEOS-based materials [17,35]. Still, it was noticed that the aerogels reinforced with recycled tire textile fiber and glass wool present a more clustered silica structure and larger voids (especially the first), confirming the presence of macropores.

### 3.3. Mechanical Characterization of the Composites

**Figure 7:**
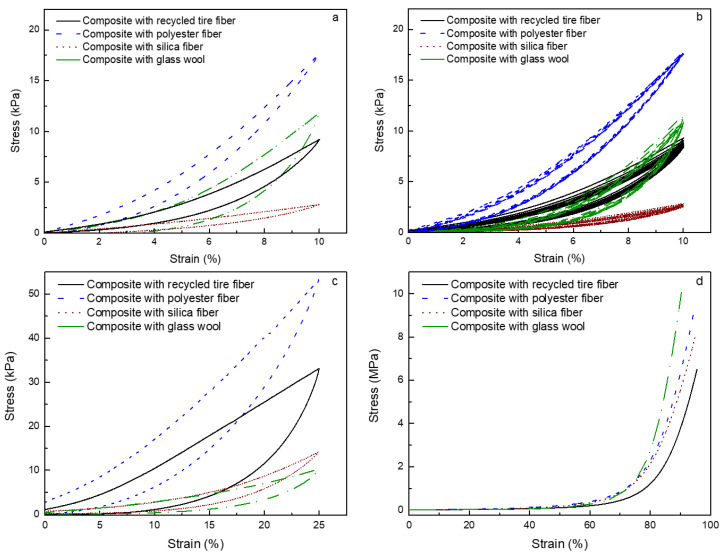
Mechanical behavior of the rubber-silica aerogel composites reinforced with different fibers. (**a**) Reversible compressive stress-strain curves of the composites until 10% strain and (**b**) ten cycles of reversible compressive stress-strain curves of the composites until 10% strain; (**c**) reversible compressive stress-strain curves of the composites until 25% strain, all with a load cell of 50 N; (**d**) uniaxial compression with a load cell of 3 kN.

The mechanical behavior of the composite materials was assessed by uniaxial compression tests, and the results are presented in Figure 7. The material’s capacity of recovery to its original shape is important for building applications, as it can regain its original shape after compression. This flexible behavior also allows to adapt better to curved surfaces. Thus, recovery tests were performed submitting the sample to 10% and 25% strain (Figure 7a,c). The results indicate an excellent behavior, as the samples are able to completely recover the original size after 10% strain (Figure 7b), and all the composites show high recoveries after 25% strain and 24 h after removing the load (Figure 7c and Table 2).

To further evaluate the capacity of the materials to withstand repeated loads, axial cyclic compression tests (10 cycles) were performed until a strain of 10% (Figure 7b). After the test, the samples only display small reductions of their initial height (Table 2). These results indicate an excellent mechanical performance of the composites, in terms of flexibility, especially if compared with pristine silica aerogels. They can withstand cyclic loads without disintegration, which is an important feature for vibration dissipation and damping.

The energy absorbing capabilities of the composite materials were also investigated by the cyclic compression tests (Table 2). The lowest energy losses were obtained for the samples reinforced with the polyester fiber blanket, indicating that only small energy dissipation occurred during the load-unload cycles. The other composites showed larger energy losses, which could be a result of some plastic deformation during the compression-decompression cycles. The plastic deformation during the compression test occurs in the silica matrix of the composite, but due to the presence of the fibers, the complete collapse of the aerogel is avoided, with the samples being able to maintain their integrity. The different values of recovery just after the test, and after 24 h, can be attributed to the slower recovery of the fibers if compared with the compression rate in which the tests are performed.

The samples were also submitted to a destructive test with the load cell of 3 kN, up to the maximum allowed force. Figure 7d presents the stress-strain curves, in which the compression progress of the samples contains three stages. At the first stage, with the strain ranging from 0% to around 30%, known as linear stage, the slope of the compression curves remains unchanged, and the open pores act as the main support of the composite. The second stage, the yielding stage (strain in the range of 30% to 60%), the stress increases at a fixed rate and the fibers become the main load-bearing part. In the final part, the densification stage (from 60% to ~95%), the collapse of the aerogel part and a significant increase in the curve slope are observed [17,36]. The samples did not recover their original height after the applied load was removed and are completely densified, as expected for the application of a compressive test on flexible samples. With the 3 kN load cell, the compression tests showed a maximum stress (Table 2) supported by all the composites in the range of 6.5–10.1 MPa, however it should be noted that these values are dependent on the maximum load of the cell.

Moreover, the dynamic stiffness was also measured for the aerogel composite with polyester fibers, as it was the sample with lowest thermal conductivity; it presented a dynamic stiffness of 11 MN·m^−3^. In comparison with other materials (e.g., recycled tire rubber: 61 MN·m^−3^, and cork/rubber composite: 184 MN·m^−3^) the measured dynamic stiffness of the new aerogel composite is significantly lower.

## 4. Conclusions

Fiber-reinforced rubber-silica aerogels have been successfully prepared and dried at ambient pressure. For the first time, aerogel composites were synthesized from a mixture of silica and recycled rubber sols and using recycled tire textile fibers, polyester fibers, silica fiber felt or glass wool as reinforcement phase. The rubber dissolution was achieved by using a peracetic acid solution, which was later used as the hydrolysis catalyst for the sol-gel process of the silica sol. As a two-step acid-base was used in this procedure, the acid solution was neutralized in the subsequent step of the synthesis (condensation) by the basic catalyst. The modification with HMDSO and TMCS was a crucial step prior drying in ambient conditions since it prevented the shrinkage of the material and allowed the expected properties of aerogel to be maintained. The addition of a recycled rubber sol into the silica sol did not lead to a negative impact in the final physical and thermal properties of the material, with the final composites with polyester fibers displaying a thermal conductivity in the superinsulation range (16.4 ± 1.0 mW·m^−1^·K^−1^). Very good results were also obtained when the silica fiber felt was used as reinforcement of the aerogel composites, being the thermal conductivity lower than 25 mW·m^−1^·K^−1^. The aerogels reinforced with the polyester blanket and inorganic fibers (silica felt and glass wool) showed an excellent thermal stability, with negligible weight loss up to 400 °C, and losses lower than 8.5 wt% up to 600 °C. The addition of polyester and inorganic fibers also improved the mechanical properties, if compared with pristine silica aerogels, with the materials exhibiting flexibility and being able to endure several axial cyclic compression tests. 

The combination of the referred properties shows that the here developed composites have a high potential to be applied as a thermal insulation material in buildings. However, for this application, the fire resistance of the material is relevant, and further work will be performed to introduce fire retardant nanoparticles in the composites, as it is well known that rubber contributes to a worse performance of the aerogel in this regard. Moreover, although the lowering of the aerogel cost is promoted by the inclusion of recycled rubber, it is also related to the price of silica precursors, and additional efforts can be done for the substitution of the alkoxide precursors by abundant silicate minerals in nature.

## Figures and Tables

**Table 1:** Key properties of the new fiber-reinforced rubber-silica aerogels.

Property	Composite with:			
	Recycled Tire Textile Fibers	Polyester Fiber Blanket	Silica Fiber Felt	Glass Wool
**Shrinkage** (%)	<1.0	<1.0	<1.0	<1.0
**Bulk density** (kg·m^−3^)	197.9 ± 6.4	115.0 ± 3.6	100.3 ± 5.3	120.1 ± 7.3
**Contact Angle** (°)	125.0 ± 5.8	131.7 ± 5.9	134.0 ± 5.6	137.5 ± 6.9
**BET specific surface area (m^2^.g^−1^)**	223.0 ± 2.9	442.4 ± 4.9	654.8 ± 6.5	204.2 ± 1.3
**BJH desorption average pore size** **(nm)**	8.3	10.4	7.4	12.0
**Thermal conductivity ^1^** (mW·m^−1^·K^−1^)	35.91 ± 0.43	24.30 ± 0.25	25.37 ± 0.06	58.05 ± 0.01
**Thermal conductivity ^2^** (mW·m^−1^·K^−1^)	28.1 ± 1.7	16.4 ± 1.0	23.2 ± 1.4	28.7 ± 1.7
**Loss of mass ^3^** (%)	42.5	3.4	1.9	0.8

^1^ Hot Disk^®^ TPS method; ^2^ Heat Flow Meter (HFM) method; ^3^ As function of temperature up to 400 °C, in N_2_ atmosphere.

**Table 2:** Mechanical properties of the new fiber-reinforced rubber-silica aerogels.

Composite Samples Reinforced with Fibers:	Young’s Modulus (kPa)	Energy Loss after		Recovery after the Test:		Recovery after 24 h:		Maximum Compression Stress ^(a)^ (MPa)
		10% Compression (%)	25% of Compression (%)	10 Cycles of 10% Compression (%)	25% of Compression (%)	10 Cycles of 10% Compression (%)	25% of Compression (%)	
**Recycled tire textile fibers**	109.5 ± 9.2	44.0 ± 0.7	59.8	89.6	78.7	96.2	94.0	6.5
**Polyester fiber blanket**	231.9 ± 16.7	15.4 ± 1.0	35.7	98.0	97.5	99.7	99.4	9.4
**Silica fiber felt**	36.9 ± 9.9	30.1 ± 1.5	38.9	91.2	96.9	97.6	97.0	8.1
**Glass wool**	138.14 ± 13.4	48.8 ± 2.7	57.6	86.5	75.6	99.8	99.2	10.1

^(a)^ Corresponds to the highest densification of the samples/maximum load of the cell.

## Data Availability

Relevant data are contained within the article. The raw/processed data required to reproduce these findings will be available upon request.

## Supplementary Materials

The following supporting information can be downloaded at: https://www.mdpi.com/article/10.3390/ma15227897/s1, Figure S1, (a) FTIR spectrum and (b) TGA of pristine rubber; Table S1, Elemental analysis of pristine rubber; Figure S2, (a) FTIR spectrum and (b) TGA of recycled tire textile fibers; Figure S3, (a) FTIR spectrum and (b) TGA of polyester fiber blanket; Figure S4, (a) FTIR spectrum and (b) TGA of silica fiber felt; Figure S5, (a) FTIR spectrum and (b) TGA of glass wool.
