# Supplementary material for: Aerogel Composites Produced from Silica and Recycled Rubber Sols for Thermal Insulation
Source: Materials (Basel). 2022 Nov 8;15(22):7897. doi: 10.3390/ma15227897 (PMC9698560; doi:10.3390/ma15227897)
Supplement: Supplementary file 1 [file materials-15-07897-s001.zip › materials-1943897-supplementary.pdf]

materials

Supporting Information

Aerogel Composites Produced from Silica and Recycled Rubber Sols for Thermal Insulation

Alyne Lamy-Mendes [1], Ana Dora Rodrigues Pontinha [1], Paulo Santos [2,*] and Luísa Durães [1,*]

[1] University of Coimbra, CIEPQPF, Department of Chemical Engineering, Rua Sílvio Lima,3030-790 Coimbra, Portugal
[2] University of Coimbra, ISISE, Department of Civil Engineering, Rua Luís Reis Santos, 3030-788 Coimbra, Portugal
* Correspondence: luisa@eq.uc.pt (L.D.), pfsantos@dec.uc.pt (P.S.)

S1. Rubber Characterization

The FTIR spectrum of the recycled rubber sample is presented in Figure S1a. By analyzing the results, it is most likely that the recycled rubber is composed by a mixture of different polymeric basis, such as Natural Rubber (NR) and Styrene-Butadiene Rubber (SBR). The spectrum shows different bands that can be attributed to: (1) stretching of N-H (Protein of NR); (2) stretching of C-H groups from the C=C-H structural unit; (3) asymmetric stretching of $-CH_3$; (4) and (5) symmetric stretching of CH_2 and CH_3 groups in natural rubber (NR); (6) amide I: stretching of C=O of the structural group R_1-(C=O)-NH-R_2; (7) amide II: in plane bending of N-H and stretching of C-N; (8) deformation of -CH_2- and rocking of -CH_3; (9) asymmetric deformation of -CH_3; (10) symmetric stretching of C-O-C and twisting of -CH_2-; (11) symmetric C-S-C group stretching vibrations in the two C-S bonds; (12) 1,4 trans-butadiene units; (13) 1,2 butadiene units; (14) and (15) out of plane bending vibration of aromatic C=C-H and C=C groups of polystyrene in SBR; and/or (15) monosulphide bonds [1–5]. The bands numbered as 1, 6, and 7 are attributed to non-isoprene compounds of NR, with the (6) -amide I and (7) -amide II bands being linked to the protein content of NR [2].

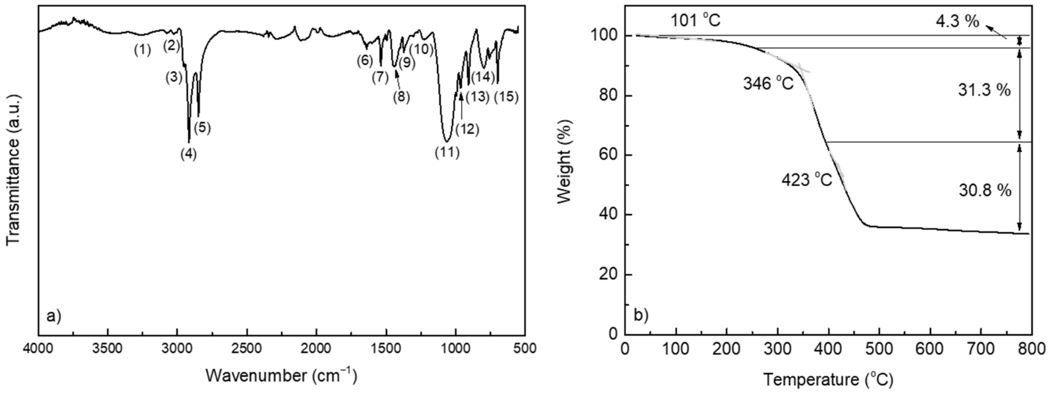

Figure S1. (**a**) FTIR spectrum and (**b**) TGA of pristine rubber.

The analysis of chemical characterizations, FTIR (Figure S1a) and elemental analysis (Table S1), of the recycled rubber allowed the confirmation of the presence of sulfur in the rubber in a non-negligible amount, which indicates that the rubber suffered a vulcanization process, as expected, resulting in a very stable material.

Table S1. Elemental analysis of pristine rubber.

Sample	%C (w/w)	%N (w/w)	%H (w/w)	%S (w/w)
**Rubber**	83.230 ± 0.736	0.582 ± 0.024	8.062 ± 0.109	1.160 ± 0.083

Thermal characterizations were also performed in the recycled rubber. From the results of TGA, it was possible to observe that the rubber has a good thermal stability, Figure S1b. Until 250 °C, only a weight loss of 4.3% was detected, and is probably the thermal degradation of remaining plasticizer portions. Two other decompositions can be observed, which agree with the results of FTIR, as they suggest a combination of NR and SBR. The first decomposition has an onset temperature of 346 °C, and a total weight loss of 31.3%, which is probably the degradation of the natural rubber. The second decomposition starts at around 400 °C and presents an onset temperature of 423 °C and weight loss of 30.8%. This second stage can be the thermal degradation of the SBR portion of the recycled tire rubber.

The thermal conductivity of the granular rubber packed bed (<0.8 mm of granules size) was also assessed, and a result of 91.09 ± 0.13 $mW.m^{-1}.K^{-1}$ (Hot Disk) was obtained. This value is quite high, especially if compared with the ones usually obtained for insulation materials, such as mineral wool (~34–45 $mW.m^{-1}.K^{-1}$), glass wool (~31–43 $mW.m^{-1}.K^{-1}$), and expanded polystyrene (EPS) (~29–55 $mW.m^{-1}.K^{-1}$) [6], so the amount of rubber used in the composites should be limited in order to control the increase of the composites' thermal conductivity.

S2. Fibers' Characterization

S2.1. Recycled Tire Textile Fiber

FTIR analysis was also performed in the recycled tire textile fiber, and the spectrum is presented in Figure S2a. The spectrum shows different bands that can be attributed to: (1) hydroxyl groups; (2) stretching vibration of hydrogen bonds in amide II and/or -NH-stretching; (3) and (4) aliphatic C-H stretching; (5) stretching of C=O of carboxylic acid group; (6) bending vibrations in hydrogen bonds of amide in mode I and/or -C=O stretching; (7) bending vibrations in hydrogen bonds of amide in mode II and/or combined N-H deformation and C-N stretching vibration modes; (8) -CH_2- scissors vibration; (9) -CH_2- scissors vibration and/or para-substituted benzene ring; (10) -CH_3 group deformation; (11) -CH_2 wagging; (12) (C=O)-C stretching of ester group; (13) and (14) O–CH_2 stretching; (15) Para-substituted benzene ring; (16) trans O–CH_2 stretching; (17) para-substituted benzene ring and/or out of plane vibration of aromatic C-H; (18) trans CH_2 rocking; (19) C=O + CCO bending and/or vibrations of adjacent two aromatic H in *p*-substituted compounds; (20) interaction of polar ester groups and benzene rings and/or bending of aromatic C-C ; (21) and (22) are assigned to the torsion in hydrogen bonds of the mode I and mode II of amide, respectively [7–13].

After analysis of the FTIR results, it was possible to confirm the presence of two main types of fibers: (1) a polyester fiber—poly(ethylene terephthalate) (PET) fiber, and (2) a polyamide fiber—nylon 6,6. The bands associated with the polyester fibers in the FTIR spectra are 1, 3, 5, 9, 11–20, while for the polyamide, the bands are 2, 4, 6–10; 21, 22. These findings are in agreement with the literature, as PET and nylon 6,6 are the most commonly used polymers in tires [14–16]. However, other fibers such as glass or rayon can also be present in the textile fiber used, but in lower amounts than the ones detected by FTIR analysis.

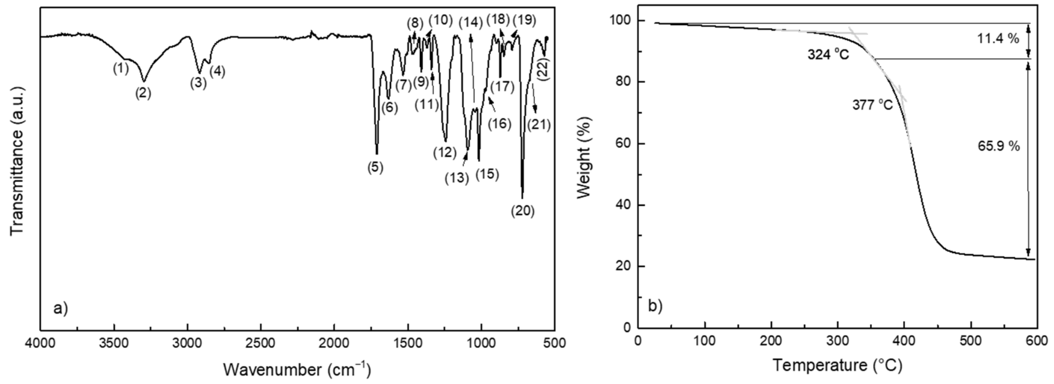

Figure S2. (**a**) FTIR spectrum and (**b**) TGA of recycled tire textile fibers.

The TGA analysis (Figure S2b) of the recycled tire textile fibers also indicates a mixture of textile fibers, as detected in FTIR, as a two-step process was observed. However, for both fibers found in the chemical characterization (PET and Nylon 6,6), higher onset temperatures were expected (around 400 °C [12,13,17–19]). The first degradation step consisted in a weight loss of 11.4% with an onset temperature of 324.3 °C, and the second step showed a weight loss of 66 % and onset temperature of 377 °C, with a maximum slope at 417 °C. It is possible that the first degradation step is related to the other fibers present in the mixture, such as rayon fiber, that presents a thermal degradation temperature between 250 °C and 350 °C [20], while the second step is attributed to the degradation of both polyester and polyamide fibers.

The thermal conductivity of this fiber mat is 67.50 ± 0.14 $mW.m^{-1}.K^{-1}$ (Hot Disk). This result is lower than the one obtained for the rubber (91.09 ± 0.13 $mW.m^{-1}.K^{-1}$), but it is still higher than common materials used for thermal insulation, as previously described.

S2.2. Polyester Fiber Blanket

The polyester fiber blanket was also submitted to FTIR analysis, and the spectrum is presented in Figure S3a. The spectrum shows different bands that can be attributed to: (1) hydroxyl groups; (2) aromatic C-H stretching; (3) and (4) aliphatic C-H stretching; (5) stretching of C=O of carboxylic group; (6) and (7) interplane skeletal vibrations of the aromatic ring; (8) CH_2 bending; (9) para-substituted benzene ring and/or in plane deformation of aromatic C-H and/or stretching of aromatic C-C; (10) CH_2 wagging; (11) (C=O)-C stretching of ester group; (12) para-substituted benzene ring and/or or in plane bending of aromatic C-H; (13) and (14) O–CH_2 stretching; (15) para-substituted benzene ring; (16) trans O–CH_2 stretching; (17) para-substituted benzene ring and/or out of plane vibration of aromatic C-H; (18) trans CH_2 rocking; (19) C=O + CCO bending and/or vibrations of adjacent two aromatic H in *p*-substituted compounds; (20) interaction of polar ester groups and benzene rings and/or bending of aromatic C-C [7–10]. After analysis of the FTIR results, it was confirmed that the polyester fiber used in this work is a poly(ethylene terephthalate) (PET) fiber.

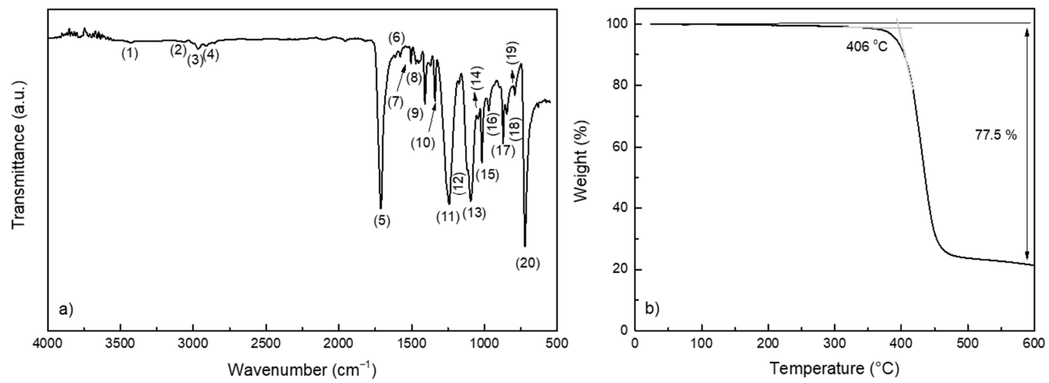

Figure S3. (**a**) FTIR spectrum and (**b**) TGA of polyester fiber blanket.

The thermal stability of the polyester fibers was investigated using TGA measurement, Figure S3b. The TGA data showed an onset temperature of 406 °C, and total weight loss of 77.5%, which is in agreement with the literature data for PET samples [17–19]. The thermal conductivity of the PET mat was also assessed, with a value of 33.90 ± 0.05 mW.m^{-1}.K^{-1} (Hot Disk). This result is much lower than the ones obtained for the rubber and the recycled tire rubber, however, it is still higher than the very low thermal conductivities of silica aerogels, that are typically in the order of 15 mW.m^{-1}.K^{-1}, at ambient temperature, pressure and relative humidity [6].

S2.3. Silica Fiber Felt

The silica fiber felt was also analyzed by FTIR, and the spectrum is presented in Figure S4a. The spectrum of this inorganic fiber shows different bands that can be attributed to: (1) silanol groups; (2) stretching of aliphatic C-H groups; (3) asymmetric bending of C-H groups; (4) and (5) asymmetric stretching vibration of Si-O-Si groups; and (6) Si-O symmetric stretching vibration [21–23]. The C-H groups are due to a thin coating of varnish on the top of the fiber felt.

The thermal degradation of the silica fibers was assessed through thermal gravimetric analysis. As observed in Figure S4b, this fiber only presented one small weight loss (around 3.9%), with an onset temperature of 270 °C, that is attributed to the degradation of the finishing organic coating. The silica fiber felt presented a thermal conductivity of 29.08 ± 0.20 mW.m^{-1}.K^{-1} (Hot Disk), the lowest between the fibers used in this work, however, as already mentioned, it is still higher than the values obtained for the silica aerogels [6].

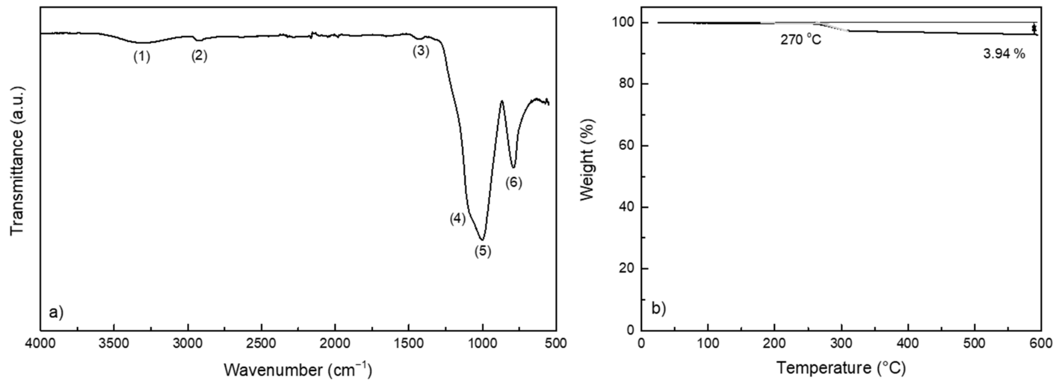

Figure S4. (**a**) FTIR spectrum and (**b**) TGA of silica fiber felt.

S2.4. Glass Wool

FTIR analysis was also performed for glass wool, and the spectrum is presented in Figure S5a. The spectrum of the glass wool shows similar bands to the ones obtained for the silica felt as expected, since both materials are mainly composed by SiO_2, and these bands can be attributed to: (1) stretching of C=C; (2) deformation vibration of C-H; (3) symmetric deformation vibration of C-H; (4) asymmetric stretching vibration of Si-O-Si groups; (5) in-plane stretching vibration of Si-O groups; and (6) Si-O symmetric stretching vibration [21–24].

Regarding the thermal stability of glass wool, the weight loss of around 35% observed in the TGA (Figure S5b) is probably due to the evaporation of adsorbed water and the degradation of organic compounds added/adsorbed in the sample during the manufacturing process [24,25]. This also explains the C-H bonds found in the FTIR spectrum. The thermal conductivity of the packed glass wool was also measured, and the sample had a value of 56.71 ± 0.03 $mW.m^{-1}.K^{-1}$ (Hot Disk).

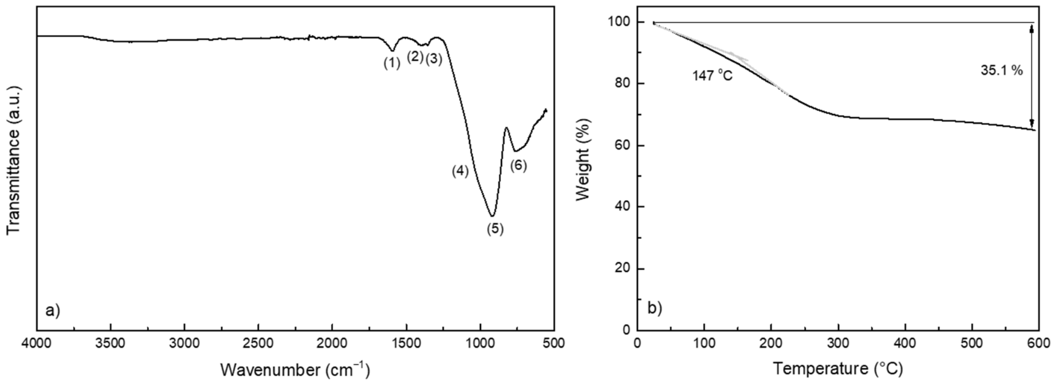

Figure S5. (**a**) FTIR spectrum and (**b**) TGA of glass wool.

References

1. Luna, C.B.B.; Araújo, E.M.; Siqueira, D.D.; de Morais, D.D.S.; Filho EA dos, S.; Fook, M.V.L. Incorporation of a recycled rubber compound from the shoe industry in polystyrene: Effect of SBS compatibilizer content. *J. Elastomers Plast.* **2020**, *52*, 3–28.
2. Rolere, S.; Liengprayoon, S.; Vaysse, L.; Sainte-Beuve, J.; Bonfils, F. Investigating natural rubber composition with Fourier Transform Infrared (FT-IR) spectroscopy: A rapid and non-destructive method to determine both protein and lipid contents simultaneously. *Polym. Test.* **2015**, *43*, 83–93.
3. Burgaz, E.; Gencoglu, O.; Goksuzoglu, M. Carbon black reinforced natural rubber/butadiene rubber and natural rubber/butadiene rubber/styrene-butadiene rubber composites: Part I: Rheological, mechanical and thermomechanical properties. *Res. Eng. Struct Mater.* **2019**, *5*, 233.
4. Gunasekaran, S.; Natarajan, R.K.; Kala, A. FTIR spectra and mechanical strength analysis of some selected rubber derivatives. *Spectrochim Acta Part A Mol. Biomol. Spectrosc.* **2007**, *68*, 323–330.
5. Garcia, P.S.; De Sousa, F.D.B.; De Lima, J.A.; Cruz, S.A.; Scuracchio, C.H. Devulcanization of ground tire rubber: Physical and chemical changes after different microwave exposure times. *Express Polym. Lett.* **2015**, *9*, 1015–1026.
6. Aegerter, M.A.; Leventis, N.; Koebel, M.M. *Aerogels Handbook*; Springer Science & Business Media, New York , USA: 2011.
7. Djebara, M.; Stoquert, J.P.; Abdesselam, M.; Muller, D.; Chami, A.C. FTIR analysis of polyethylene terephthalate irradiated by MeV He+. *Nucl. Instrum. Methods Phys. Res. Sect. B Beam. Interact. Mater. Atoms* **2012**, *274*, 70–77.
8. Edge, M.; Wiles, R.; Allen, N.S.; McDonald, W.A.; Mortlock, S.V. Characterisation of the species responsible for yellowing in melt degraded aromatic polyesters—I: Yellowing of poly (ethylene terephthalate). *Polym. Degrad. Stab.* **1996**, *53*, 141–151.
9. Holland, B.J.; Hay, J.N. The thermal degradation of PET and analogous polyesters measured by thermal analysis–Fourier transform infrared spectroscopy. *Polymer* **2002**, *43*, 1835–1847.
10. Pereira AP dos, S.; Silva MHP da Lima Júnior, É.P.; Paula A dos, S.; Tommasini, F.J. Processing and characterization of PET composites reinforced with geopolymer concrete waste. *Mater. Res.* **2017**, *20*, 411–420.
11. Farias-Aguilar, J.C.; Ramírez-Moreno, M.J.; Téllez-Jurado, L.; Balmori-Ramírez, H. Low pressure and low temperature synthesis of polyamide-6 (PA6) using Na0 as catalyst. *Mater. Lett.* **2014**, *136*, 388–392.

12. Zhang, M.; Gao, Q.; Yang, C.; Pang, L.; Wang, H.; Li, R.; Xing, Z.; Hu, J.; Wu, J. Preparation of antimicrobial MnO_4^--doped nylon-66 fibers with excellent laundering durability. *Appl. Surf. Sci.* **2017**, *422*, 1067–1074.
13. Charles, J.; Ramkumaar, G.R.; Azhagiri, S.; Gunasekaran, S. FTIR and thermal studies on nylon-66 and 30% glass fibre reinforced nylon-66. *E-J. Chem* **2009**, *6*, 23–33.
14. Mahdavipour, Z.; Karimi, M.; Hoseini, S.A. Study of thermomechanical properties of hybrid tire cord. *Polym. Adv. Technol.* **2020**, *31*, 2250–2258.
15. Camlibel, N.O. *Polyester: Production, Characterization and Innovative Applications*; IntechOpen. London, United Kingdom: 2018.
16. Czajczyńska, D.; Krzyżyńska, R.; Jouhara, H.; Spencer, N. Use of pyrolytic gas from waste tire as a fuel: A review. *Energy* **2017**, *134*, 1121–1131.
17. Gashti, M.P.; Navid, M.Y.; Rahimi, M.H. Effects of coating of nano-and microemulsion silicones on thermal properties and flammability of polyethylene terephthalate textile. *Pigment. Resin. Technol.* **2013,** *42*, 34-44.
18. Butnaru, I.; Serbezeanu, D.; Bruma, M.; Sava, I.; Gaan, S.; Fortunato, G. Physical and thermal properties of poly (ethylene terephthalate) fabric coated with electrospun polyimide fibers. *High. Perform. Polym.* **2015**, *27*, 616–624.
19. Khalaf, E.; Awad, S. Improvement of chemical and thermal properties of polyethylene terephthalate (PET) by using multi-walled carbon nanotubes (MWCNTs). *Int. J. Mater. Sci. Appl.* **2016**, *5*, 297–301.
20. Peng, S.; Shao, H.; Hu, X. Lyocell fibers as the precursor of carbon fibers. *J. Appl. Polym. Sci.* **2003**, *90*, 1941–1947.
21. Bonon, A.J.; Weck, M.; Bonfante, E.A.; Coelho, P.G. Physicochemical characterization of three fiber-reinforced epoxide-based composites for dental applications. *Mater. Sci. Eng. C* **2016**, *69*, 905–913.
22. Al-Oweini, R.; El-Rassy, H. Synthesis and characterization by FTIR spectroscopy of silica aerogels prepared using several Si (OR) 4 and R″ Si (OR′) 3 precursors. *J. Mol. Struct* **2009**, *919*, 140–145.
23. Sakai, S.; Yamaguchi, T.; Putra, R.A.; Watanabe, R.; Kawabe, M.; Taya, M.; Kawakami, K. Controlling apatite microparticles formation by calcining electrospun sol–gel derived ultrafine silica fibers. *J. Sol.-Gel. Sci. Technol.* **2012**, *61*, 374–380.
24. Dastorian Jamnani, B.; Hosseini, S.; Shavandi, A.; Hassan, M.R. Thermochemical properties of glass wool/Maerogel composites. *Adv. Mater. Sci. Eng.* **2016**, *2016*, 6014874.
25. Lemougna, P.N.; Yliniemi, J.; Nguyen, H.; Adesanya, E.; Tanskanen, P.; Kinnunen, P.; Roning, J.; Illikainen, M. Utilisation of glass wool waste and mine tailings in high performance building ceramics. *J. Build. Eng.* **2020**, *31*, 101383.
